# Supplementary material for: Systematic framework to assess social impacts of sharing platforms: Synthesising literature and stakeholder perspectives to arrive at a framework and practice-oriented tool
Source: PLoS One. 2020 Oct 8;15(10):e0240373. doi: 10.1371/journal.pone.0240373 (PMC7544048; doi:10.1371/journal.pone.0240373)
Supplement: S1 Appendix — (DOCX) [file pone.0240373.s001.docx]

# S1 Appendix. Keywords Identified During Preliminary Literature Review

**1st Round Keywords (25)**

Public perception

Cultural capital

Inequality

Openness

Gig labor

Marginal population

Regulation

Collective efficacy

Satisfaction

Self-expression

Belonging

Community

Trust

Cooperation

Adoption

Motive

Participation

Social commerce

Interests

Power structure

Social cohesion

Inclusivity

Justice

Empowerment

Conflict

**2nd Round Keyword (25):**

Digital inequality

Governance

Deregulation

Community governance

Public power

Economic opportunity

Sustainable society

Sharing for community

Sharing for charity

Casual sociability

Social capital

Cultural capital

Cosmopolitan capital

Financial democracy

Financial inclusivity

Human rights

Sociality

Reciprocity

Belonging

Civic engagement

Democratic legitimacy

E-governance

Motivations

Employment

Convenience
