# Supplementary material for: Systematic framework to assess social impacts of sharing platforms: Synthesising literature and stakeholder perspectives to arrive at a framework and practice-oriented tool
Source: PLoS One. 2020 Oct 8;15(10):e0240373. doi: 10.1371/journal.pone.0240373 (PMC7544048; doi:10.1371/journal.pone.0240373)
Supplement: S2 Appendix — (DOCX) [file pone.0240373.s002.docx]

# S2 Appendix. Social Aspects and Impacts from Preliminary Literature Review

**Table A.** Social aspects and impacts identified in the preliminary literature review

| **Social Aspect** | | **Social Impacts** |
| --- | --- | --- |
| **Empowerment** | | - Personal identity and empowerment - Freedom liberty - Economic empowerment - Employment and unemployment |
| **Trust Among Strangers** | | - Social interactions - Building community - Authenticity of individuals over review systems |
| **Inclusivity** | Builds social Capital | - Learning and teaching - New social ties and networks - Skills, knowledge and competences - Education and training |
|  | Social Cohesion | - Building community - Social integration - Community cohesion - Making friends - Eating, cooking and growing together - Mutual learning and teaching |
|  | Social Inclusion | - Social inclusion/exclusion - Discrimination - Join to avoid competition with existing systems - Sufficiency or lack of political and regulatory frameworks - Citizen participation and democracy |
| **Social Justice** | | - Privacy, safety and security - Equality, equity - Users’ rights (consumer and provider) - Equal opportunities |
