# Supplementary material for: Systematic framework to assess social impacts of sharing platforms: Synthesising literature and stakeholder perspectives to arrive at a framework and practice-oriented tool
Source: PLoS One. 2020 Oct 8;15(10):e0240373. doi: 10.1371/journal.pone.0240373 (PMC7544048; doi:10.1371/journal.pone.0240373)
Supplement: S3 Appendix — (DOCX) [file pone.0240373.s003.docx]

# S3 Appendix. Definitions of Social Aspects Presented at Workshop

**Empowerment**

Empowerment is “the act or action of empowering someone or something: the granting of the power, right, or authority to perform various acts or duties”. With information technology, individuals gain digital empowerment which includes better networking, communication and cooperation opportunities, and increased competence of individuals and communities to act as influential participants in the society. In the sharing economy, both peer providers and peer users are empowered. Peer providers are empowered as they can unlock the value of their idling assets, time and skills and can thereby earn additional income. Peer users are empowered as they gain temporary access to assets they need but cannot afford otherwise. Peer providers and peer users are also empowered if they have influence over how the platform operates or how the city develops its governance frameworks.

**Trust**

Trust is assured reliance on the character, ability, strength, or truth of someone or something. It plays an essential role in the formation of interactions and relationships in the sharing economy, where three types of trust can be distinguished: trust in peers (providers and users), trust in platform and trust in products or service.

**Inclusivity**

Inclusivity is the quality of trying to include many different types of people and treat them all fairly and equally, who might otherwise be excluded or marginalized, such as vulnerable groups, e.g. handicapped or learning-disabled, or racial and sexual minorities. Inclusivity refers to a sense of shared ownership or join vision to contribute to the sustainability of a city. Inclusion emphasises the need for broader consultation and engagement of communities in decision making.

**Social Justice**

Justice is the quality of being just, impartial, or fair. Sharing platforms may have access to the personal data about users and providers. This may raise concerns regarding privacy, safety, security, and rights. Sharing platforms may have very different impacts on different social groups, e.g., based on age, gender, religion, ethnicity and nationality. Thus it is important to assess impacts of the already established platform companies and initiatives. This creates the need for the platforms to open their data for research purposes. For this we need governance and regulatory measures to prevent and mitigate negative impacts of the sharing economy and to enable data to be used for societal purposes.
