# Supplementary material for: Systematic framework to assess social impacts of sharing platforms: Synthesising literature and stakeholder perspectives to arrive at a framework and practice-oriented tool
Source: PLoS One. 2020 Oct 8;15(10):e0240373. doi: 10.1371/journal.pone.0240373 (PMC7544048; doi:10.1371/journal.pone.0240373)
Supplement: S4 Appendix — (DOCX) [file pone.0240373.s004.docx]

# **S4 Appendix. Workshop data**

# **Group 1 (Company)**

## Trust (1)

- Definition: integrity, Mutual trust/deal.
- Measurable indicators: rating system; selling information; returning customers, communication; transparency.

## Inclusivity (a shared 2)

- Definition: participation; co-decision; justice; shared vision/philosophy; being seen (in a positive way, listen and include).
- Measurable indicators: survey; returning customers; rating complaints.

## Social justice (a shared 2)

- Definition: fairness; non-judgemental approach; inclusivity; privacy.
- Measurable indicators: n/a.

## Empowerment (3)

- Definition: The feeling of being ”lifted” and assured you are part of something bigger.
- Measurable indicators: volume in; money made; survey.

# Group 2 (Interest group + Authorities)

# Case: to help housing associations to arrange “sharing hubs” for garden tools, drills etc.

## Empowerment (1)

- Definition: for the providers this is to appoint a responsible person for maintenance of sharing hubs; create rules; for the users this is to avoid having the responsibility for the maintenance; avoid expensive investments; avoid having the trouble when the product becomes waste.
- Measurable indicators: How many use the service (only those who feel that they have some utility from using the service).

## Trust (2)

- Definition: Rules create trust for the system; follow rules without direct control (instead using social control).
- Measurable indicators: Measured by considering how the rules are followed and by considering: how many things have disappeared; and how many things are broken.

## Inclusivity (not specified)

- Definition: All who live in the housing association can join; the association does things that benefit all (the shape of the association influences this).
- Measurable indicators: Ensures that other requirements on access are also including in the sharing hub.

## Social justice (not specified)

- Definition: Registry of borrowers -> only names; closed group reduce the need to control. Less risk of miss-use of personal data.
- Measurable indicators: Prevent that some persons over-use the tools; measure how the distribution looks.

Extra comment on the paper sheet from this group: “Possible development: offer services/knowhow for example using a drill.”

# Group 3 (Interest group)

## Empowerment (1)

- Definition: Knowledge and skills; channel and platforms to have a voice; ownership.
- Measurable indicators: Gained knowledge and skills; access to and usage of channels and platforms; sense of ownership; number of reduced new actions within empowerment that have been added in the project during the project time.

## Trust (2)

- Definition: Need to be trust-worthy (build on science, know impacts etc).
- Measurable indicators: Environmental, social, economic impacts; measure transparency.

## Inclusivity (3)

- Definition: adapt to others; to strive to have those groups which normally are not feeling involved are also included.
- Measurable indicators: how much is the participation increasing in under-represented groups.

## Social justice (4)

- Definition: should be including so that the entire society is included. This will have as a side effect that inclusivity, trust and empowerment increase in this context.
- Measurable indicators: how much is the participation increasing in under-represented groups.

# Group 4 (municipality)

## Empowerment (not specified)

- Definition: The participation of citizens in the processes in an early stage; voicing their opinions; involving.
- Measurable indicators: Different groups representation in the preparatory work for decisions. (“to get out of the facebook bubble”)

## Trust (not specified)

- Definition: Trust is something that you can earn, not demand; Trust can disappear fast; transparency for the service between persons.
- Measurable indicators: Ranking systems on platforms; platforms in general, ranking in both directions, creating a transparency; objective (thirdparty) evaluation institutes.

## Inclusivity (not specified)

- Definition: Reach those are not interested. Whose role to take responsibility for exposed groups? the role of the municipality to listen to all groups who use services; not using but also the right way; participatory that more should use services; legislation.
- Measurable indicators: State and municipality give direction so that all should use; adjustment for all for sharing services.

## Social justice (not specified)

- Definition: services shall only be used when users need them, and no unnecessary storage; consumer information.
- Measurable indicators: national and international control (auditing?) organisations; municipality and state to require XXX data.

# Group 5 (Municipality)

## Social justice (1)

- Definition: Education and knowledge; acknowledge unfairness; long-term social sustainability.
- Measurable indicators: Level of participation; knowledge level; concrete measures showing that problems are addressed seriously, for example improving housing areas, meeting places for sharing where it is really needed.

## Empowerment (2)

- Definition: Influence; participation; economic indecency.
- Measurable indicators: The possibility for citizens to suggest, dialogue meetings; level of participation in local meeting places; fewer receiving social support.

## Trust (3)

- Definition: Trust (technology and social); safety; municipality as a warrant (?) (instutionalization of apps).
- Measurable indicators: Level of participation/visits; deep interviews.

## Inclusivity (4)

- Definition: Access including language; user friendliness; communication.
- Measurable indicators: Focus groups (deep interviews, design); education (participation groups indicating socioeconomic profiles or for example educational level); number of languages; open hours.

# Group 6 (Municipality)

## Empowerment (not specified)

- Definition: The possibility to meet other persons in the city and to shape the city - > a shift in power from public and commercial interests to civil society; “meet, participate, influence”.
- Measurable indicators: Meeting places (number and access); perceived possibility to influence.

## Inclusivity (not specified)

- Definition: Not the same (as the above?) but of similar/equal value.; the seven grounds for discrimination; digital can exclude (isolation and loneliness can contribute to reduced trust).
- Measurable indicators: Offers services and alternatives to digital use; how use the services in different groups (older, disabled etc.).

## Social justice (not specified)

- Definition: Space for different groups to take the initiative based om their needs; groups with less resources can not fulfill their needs.
- Measurable indicators:

## Trust (not specified)

- Definition: Same as in the guiding document; high trust in the public sector; low trust in companies and unknown persons; can a high level of trust to the public sector be an obstacle for citizen-driven development?
- Measurable indicators: perceived safety; propensity to lend things to friends, neighbors, acquaintances; number of loans.

# Group 7 (Authorities merged with G2)

## Empowerment (1)

- Definition: (egenmakt and bemyndigande). Give power and possibility, the right and possibility; to fill someone with power.
- Measurable indicators: n/a

## Trust (2)

- Definition: credibility, trust, mutual trust, transparency, trust through standards and certifications.
- Measurable indicators: High usage 🡪 high trust; High measured complaints 🡪 low trust; satisfied customers 🡪 high trust; very system dependent for example trust of Airbnb in systems/platforms; high level of problem with the platform 🡪 low trust.

## Inclusivity (not specified)

- Definition: n/a
- Measurable indicators: n/a

## Social justice (not specified)

- Definition: n/a
- Measurable indicators: n/a

# Group 8 (Authorities)

## Trust (1)

- Definition: The bigger the economic instability, the higher level of trust is needed.
- Measurable indicators: Ranking or similar for private/non-profit based initiatives; organisations/companies can guarantee the function.

## Social justice (2)

- Definition: Sharing should and can be a part of creating a more equal society; today there is a certain degree of status in the sharing which can counteract equality in the participation.
- Measurable indicators: n/a

## Inclusivity (3)

- Definition: Closely related to trust, and it is easier to participate in a group that you can identify yourself with.
- Measurable indicators: n/a

## Empowerment (4)

- Definition: Freedom; independence; status (but also the opposite)
- Measurable indicators: n/a

# Group 9 (University)

## Trust (1)

- Definition: n/a
- Measurable indicators: Trust to others (peers): recommendations in relation to number of transactions; how much are rule and structures followed; trust to the platform: number of users, well-functioning communication (contact chanel, help for problem solving (personal!), control/follow up of structures/codes of conduct; trust to products /services: how well is the experiences matching the provided information; number of times of usage.

## Inclusivity (2)

- Definition: n/a
- Measurable indicators: How well is the information reaching out (language etc.)?; how well are different groups represented (background, age, gender, socioeconomic profiles, education etc.); how much have they been part of the design; are different groups in society targeted in communication; listen to different users’ opinions and experiences and what they can contribute with.

## Social justice (not specified)

- Definition: n/a
- Measurable indicators: criteria/targets for how social and exposed groups should take part of the service; degree of profit motive; how is the profit shared between the users; transparency; access.

## Empowerment (not specified)

- Definition: Perceived feeling of being able to influence and have influence in questions related to decisions, access to resources and participation.
- Measurable indicators: Access to new forums (?) and resources; degree of possibility to influence for example the platform; be an active part of a network; social capital.

**1. Trust is:**

*For providers or the platform*

Integrity and mutual trust in deal (G1: Company), being able to follow rules without direct control (instead using social control) (G2: Interest), trust-worthy information regarding impacts etc. (G3: Interests), robust level of economic stability (G8: Authority).

*For users or society*

Trust is something that you can earn not demand, trust can disappear quickly, transparency of the service among people (G4: Municipality), trust in technology and society, institutionalisation of apps (G5: Municipality), credibility, mutual trust, transparency through standards and certifications (G7: Authority)

**How to measure**

*For providers or the platform*

- Presence of a rating system (G1)
- Availability of seller’s (provider’s) information (G1)
- Transparency of the platform and communication (G1)
- Measure of to what extent the rules are followed (G2)
- Transparency about the environmental, social and economic impacts (G3)
- Evaluation of the ranking systems of the platform by third party (G4)
- Trust in the platform: number of users, well-functioning communication (contact channel, help for problem solving); control/follow-up of structures/codes of conduct (G9)
- Trust in products and services: Extent to which the experiences are matching with the provided information; number of times of usage (G9)

*For users or society*

- How many assets/things are lost/disappeared or got broken (mishandled) (G2)
- Level of participation(visits) through interviews (G5)
- Survey of perceived safety over the platform (G6)
- Survey of propensity to lend things to friends, neighbours, acquaintances (G6)
- Number of loans (G6)
- Survey of frequency of usage as a measure of high level of trust; high volume of complaints as a measure of low trust; customer satisfaction as measure of trust; problems with the platform (G7)
- Trust to others (peers): number of recommendations with respect to number of transactions; To what extent the rules and structures are followed (G9)

**2. Empowerment is:**

*For providers or the platform*

“The feeling that you are part of something big (G1: Company) and are able to create rules (G2: Interest group), platform have knowledge and skills, and a voice and ownership (G3: Interest)”

*For users or society*

To avoid having the responsibility for the investment, maintenance, and trouble of disposal of the product at its end-of-life (G2: Interest/Authority) participation of citizens in the processes in an early stage so that they can voice their opinions through participation (G4 Municipality) and influence (G5: Municipality), the possibility to meet other people and to shape the city, a shift in power from public and commercial interests to civil society (G6: Municipality), to grant users power to influence, perceived feeling of being able influence in question related to decisions, and access to resources and participation (G9: University)

**How to measure**

*For providers or the platform*

- Survey of the amount of earnings (G1)
- Number of people using the service (those who feel the utility of the offered service) (G2)
- Acquired knowledge and skills, access to platform, sense of ownership (G3)
- Number of initiative concerning empowerment that have been added and addressed during the project’s timeline (G3)

*For users or society*

- Different groups represented in the preparatory work for decisions (G4)
- Possibility of citizens to suggest or participate in dialogue, level of participation in meetings (G5)
- Reduced reliance on social support (G5)
- Perceived possibility to influence decisions (society) (G6)
- Access to new forums and resources (G9)
- Degree of possibility to influence e.g. the platform (G9)
- Active participation in a network (G9)

**3. Inclusivity is:**

*For the providers or the platform*

Equal participation in decision-making and being visible and inclusive (G1: Company), everyone can join and doing things that benefit all (G2: Interest/Authority), adapt to groups that are normally don’t feel involved (G3: Interest), to reach out to all groups who are using the service as well as not using (G4: Municipality), accessible (in all languages, user-friendliness and communication ) (GG5: Municipality) , equal value to the seven grounds of discrimination (sex, transgender, religion or other belief, disability, sexual orientation, age) (G6: Municipality)

*For users or society*

easier to participate in a group that one can easily identify with (G8: Authority)

**How to measure**

- Survey of returning customers, rating complaints (G1: Company)
- Survey of increasing trend in the participation in under-represented groups (G3: Interest)
- Focus groups / deep interviews with groups belonging to different socio-economic groups (G5: Municipality)
- If organisations have open hours (G5: Municipality)
- Survey of use of service by different group (older, disabled etc.) (G6: Municipality)
- Analysis of: how well the information is reaching out (language etc.); How well different groups are represented; to what extent they have been part of the design; Are different part of the society targeted in communication; are opinions and experiences of different users and how can they contribute heard (G9: University)

**Social justice is:**

Fair processes and non-judgemental approach and respecting privacy issues (G1: Industry); less risk of misuse of users’ personal data (G2: Interest/Authority), should include the entire society (G3: Interest) ; acknowledging unfairness, long-term social sustainability (G5: Municipality); space for different groups to take initiatives based on their needs (G6: Municipality), sharing should and can be a part of the creating a more equal society (G8: Authority)

**How to measure**

- How the participation has increased in under-represented groups (G2: Interest/ Authority)
- Presence of auditing system (national/international), sharing data with municipality and state (G4: Municipality)
- Analysis of concrete measures showing that the problems are addressed seriously. (G5: Municipality)
- Criteria/targets for how social and exposed groups should take part in the service; degree pf profit motive; how is the profit shared among the users; level of transparency and access of information (G9: University)

**Definition:**

| **Group** | **Empowerment** | **Trust** | **Inclusivity** | **Social justice** |
| --- | --- | --- | --- | --- |
| G1 | The **feeling of being ”lifted”** and assured you are **part of something bigger**. | integrity, Mutual trust/deal. | **participation**; **co-decision**; justice; **shared vision/philosophy**; **being seen** (in a positive way, listen and include). | **fairness**; **non-judgemental** **approach**; inclusivity; **privacy** |
| G2 | for the providers this is to **appoint a responsible person** for maintenance of sharing hubs; **create rules**; for the users this is **to avoid having the responsibility** for the maintenance; **avoid expensive investments**; **avoid having the trouble** when the product becomes waste. | **Rules create trust** for the system; **follow rules without direct control** (instead using social control). | **All who live in the housing association can join**; the association does **things that benefit all** (the shape of the association influences this) | **Registry of borrowers** -> **only names**; **closed group reduce the need to control**. **Less risk of miss-use of personal data** |
| G3 | **Knowledge and skills**; **channel and platforms to have a voice**; **ownership**. | Need to be **trust-worthy** (build on science, know impacts etc) | **adapt to others**; to **strive to have those groups** which **normally are not feeling involved** are also included | **should be including** so that the **entire society is included**. This will have as a **side effect** that inclusivity, trust and empowerment increase in this context |
| G4 | The **participation of citizens in the processes in an early stage**; **voicing their opinions; involving**. | Trust is something that you can **earn**, **not demand**; Trust can **disappear fast**; **transparency** for the service between persons. | **Reach those are** **not interested**. Whose **role to take responsibility for exposed groups**? the **role of the municipality to listen to all groups** who use services; not using but also the right way; **participatory that more should use services**; legislation. | services shall only be **used when users need them,** and **no unnecessary storage**; **consumer information**. |
| G5 | **Influence**; **participation; economic independency**. | **Trust** (technology and social); **safety**; **municipality as a warrant** (?) (**instutionalization** of apps). | **Access** including language; **user friendliness**; **communication** | **Education** and **knowledge**; **acknowledge unfairness**; long-term **social sustainability**. |
| G6 | The **possibility to meet other persons in the city and to shape the city** - > a **shift in power from public and commercial interests to civil society**; “**meet, participate, influence”.** | Same as in the guiding document; **high trust in the public sector**; **low trust in companies** and **unknown persons**; can a **high level of trust to the public sector be an** **obstacle for citizen-driven development**? | Not the same (as the above?) but of **similar/equal value**.; the [**seven grounds for discrimination**](http://www.do.se/other-languages/english/protected-grounds-of-discrimination/) (sex, transgender identity expression, ethnicity, religion or other belief, disability, sexual orientation, age); **digital can exclude** (isolation and loneliness can contribute to reduced trust) | **Space for different groups** to take the **initiative based on their needs**; **groups with less resources can not fulfill their needs** |
| G7 | **Give power** and **possibility**, **the right and possibility**; to **fill someone with power**. | **credibility**, **trust**, **mutual trust**, **transparency**, **trust** through **standards and certifications**. | N/A | N/A |
| G8 | **Freedom**; **independence**; **status** (but also the opposite) | The bigger the **economic instability**, the **higher level** of **trust is needed** | **Closely related to trust**, and it is **easier to participate** in a **group that you can identify yourself with** | Sharing should and can be a part of **creating a more equal society**; today there is a certain degree of status in the sharing which can counteract equality in the participation |
| G9 | **Perceived feeling of being able to influence** and **have** **influence in questions related to decisions**, **access to resources** and **participation**. | N/A | N/A | N/A |

**How to measure:**

| **Group** | **Empowerment** | **Trust** | **Inclusivity** | **Justice** |
| --- | --- | --- | --- | --- |
| G1 | volume in; **money made**; **survey** | **rating system**; **seller information**; **returning customers**, **communication**; transparency | survey; **returning customers**; **rating complaints** | N/A |
| G2 | How **many use the service** (only those who feel that they have some utility from using the service). | Measured **by considering how the rules are followed** and by considering: **how many things have disappeared**; and **how many things are broken** | Ensures that **other requirements on access are also including** in the sharing hub | Prevent that **some persons over-use the tools**; **measure how the distribution looks** |
| G3 | **Gained knowledge** and **skills**; **access to** and **usage of channels** and **platforms**; **sense of ownership**; number of reduced new actions within empowerment that have been added in the project during the project time | Environmental, social, economic impacts; **measure transparency** | how much is the **participation increasing** **in under-represented groups**. | how much is the **participation increasing** in **under-represented groups** |
| G4 | Different **groups representation** in the preparatory work for decisions. (“to get out of the facebook bubble”) | **Ranking systems** on platforms; platforms in general, **ranking in both directions**, creating a **transparency**; objective **(third party) evaluation** institutes | **State and municipality give direction** so that all should use; adjustment for all for sharing services | **national and international control** (auditing?) organisations; **municipality and state to require XXX data** |
| G5 | The **possibility for citizens to suggest**, **dialogue meetings**; level of **participation in local meeting places**; **fewer receiving social support** | Level of **participation/visits**; deep interviews | Focus groups (deep interviews, design); education (**participation groups indicating socioeconomic profiles** or for example educational level); number of languages; **open hours** | **Level of participation**; **knowledge level**; **concrete measures showing that problems are addressed seriously**, for example improving housing areas, meeting places for sharing where it is really needed |
| G6 | Meeting places (**number and access**); perceived **possibility to influence**. | **perceived safety**; **propensity to lend things** to friends, neighbors, acquaintances; **number of loans** | **Offers services and alternatives to digital use**; how use the **services in different groups** (**older, disabled** etc.) | N/A |
| G7 | N/A | **High usage** 🡪 high trust; **High measured complaints** 🡪 low trust; **satisfied customers** 🡪 high trust; **very system dependent** for example trust of Airbnb in systems/platforms; high level of problem with the platform 🡪 low trust | N/A | N/A |
| G8 | N/A | **Ranking** or similar for private/non-profit based initiatives; organisations/companies can **guarantee** the function | N/A | N/A |
| G9 | Access to new **forums** (?) and **resources**; degree of **possibility to influence** for example **the platform**; be an **active part of a network**; social capital | **Trust to others** (peers): **recommendations** in relation to **number of transactions**; how much are **rule and structures** followed; **trust to the platform**: **number of users**, **well-functioning communication** (contact chanel, help for problem solving (**personal!**), **control/follow up of structures/codes of conduct**; **trust to products /services**: how well is the **experiences matching** **the provided information**; number of **times of usage**. | **How well is the information reaching out** (language etc.)?; **how well are different groups represented** (background, age, gender, socioeconomic profiles, education etc.); **how much have they been part of the design**; **are different groups in society targeted in communication**; **listen to different users’ opinions and experiences** and **what they can contribute with**. | **criteria/targets for how social and exposed groups should take part of the service**; **degree of profit motive**; **how is the profit shared between the users**; **transparency**; **access**. |

Ranking:

| **Group** | **Empowerment** | **Trust** | **Inclusivity** | **Justice** |
| --- | --- | --- | --- | --- |
| G1 | 3 | 1 | 2 | 2 |
| G2 | 1 | 2 | - | - |
| G3 | 1 | 2 | 3 | 4 |
| G4 | - | - | - | - |
| G5 | 2 | 3 | 4 | 1 |
| G6 | - | - | - | - |
| G7 | - | - | - | - |
| G8 | 4 | 1 | 3 | 2 |
| G9 | 1 | 2 | - | - |

Overall ranking

| Empowerment | Trust | Justice | Inclusivity |
| --- | --- | --- | --- |
| 1 | 2 | 3 | 4 |
