# Supplementary material for: Systematic framework to assess social impacts of sharing platforms: Synthesising literature and stakeholder perspectives to arrive at a framework and practice-oriented tool
Source: PLoS One. 2020 Oct 8;15(10):e0240373. doi: 10.1371/journal.pone.0240373 (PMC7544048; doi:10.1371/journal.pone.0240373)
Supplement: Attachment — Submitted filename: Reviewer Responses.docx [file pone.0240373.s005.docx]

**Response to Reviewers**

Fantastic! We are so grateful to the reviewers (and the editor) for their time and attention in providing meaningful feedback on our manuscript. We are excited that they see our contribution, and look to address their feedback meaningfully in the resubmission of our manuscript. The bulk of the feedback concentrated on our *Introduction* and *Background Literature*, as opposed to our approach and output. As such, we have focused our efforts there.

We acknowledge many of the valuable points of feedback and suggestions made by the reviewers. As a result, we have spent considerable time reflecting on how to improve our manuscript and hope to please the reviewers as well as the academic editors. The reviewers highlighted several areas for improvement, which we address in our revision, including:

- Increase the necessary information provided in the *Introduction* to justify the research gap, contribution, and approach;
- Introduce key concepts earlier in the manuscript, and provide definitions to support the reader to delineate fuzzy concepts;
- Engage more critically and profoundly with background literature, both in the *Introduction* and *Background Literature* sections.

While this provides an overview of the suggestions from reviewers and the focus of our revision, we will respond to each of the points specifically. Black text signifies the reviewer responses; green text signifies our response. We provide line numbers corresponding to the revised manuscript, with all revisions highlighted for clarity.

**Reviewer #1:**
The paper aims to make an analysis of the “sharing platforms” and their social impact. The idea is attractive and novel, the document is worked and is widely documented. The sources of information have value in itself, and the goal of setting a set of measurement variables is interesting.

Thanks for the feedback. We appreciate your kind remarks and we are pleased you see value in our work.

The work deals with a lot of aspects that are very interrelated and that are generally little concretes. This makes the work very long. From my point of view, the paper must be written in a clearer and more concrete way making an effort to show the reader the relationship between the concepts managed in a profound and concrete way. The Workshop and the Table 5 seem to be especially valuable.

We agree with the reviewer's observation regarding the many interrelated terms. This was also a struggle we encountered when engaging with the relevant academic literature. While some of the fuzziness is an artefact of the existing literature, we seek to improve the organisation and elaboration of the social aspects associated with our framework. To do so, we introduce the four social aspects – trust, empowerment, inclusivity, and social justice – already in the *Introduction* **(Lines 78 – 83)**. In addition, we provide additional elaboration of these concepts in the *Background Literature*, including Table 1 with working definitions and relevant literature **(Lines 146 – 158)**. By including this information earlier, we hope to provide the reader with the necessary structure and context to navigate our manuscript.

New communication technologies (ICTs) allow for a reduction in costs that are the basis of the “sharing economy”. Technological advances in communication generate value in a transversal way and one way in which it produces value is by reducing transaction costs which increases the supply and allows access to the service at a lower price and generates social wealth since it is consumers who appropriate the value generated. The cost reduction is a consequence of: 1) increasing the level of information before and after the contract (adverse selection and moral hazard) (what at work has been called “trust”). Increased information also reduces “residual loss”, giving access to demand cheaply to the producer and giving access to more supply by the consumer; 2) reduces limited rationality, facilitating information management by grouping it into satisfaction indicators that allow for easy interpretation (likes, stars...).

When transaction costs are high, the company replaces the market as a form of concentration of resources in order to save the high costs of marketing the product. The aforementioned transaction cost reduction allows the market (individual persons) to replace the company as an allocation system, thus moving from an authority-based system (company) to a consensus-based system (market). These classic concepts can be an important reference point at work.

Thanks for this background, which was missing from our manuscript. We hear your suggestion, and have included this context now in describing the sharing economy **(Lines 108 – 117)**.

Expanding the market and reducing commercial costs allows to take better advantage of idle resources, reducing slack resources and increasing the rate of generating value (renting the house when you are not, sharing the vehicle when you don't need it, ...). These two aspects seem to favor the lower layers of society (the idea that is transmitted at work through “democratization”), improving “social justice”, ... (this must be well-argued).

This is an interesting point. One would think that the sharing economy may democratise consumption and provide greater access to resources otherwise unattainable, theoretically supporting lower layers of society. However, in practice, active users in the sharing economy tend to be younger, educated, and more affluent. Furthermore, the benefits of participation are not equally distributed, often times favouring the more wealthy and powerful (e.g. home owners are able to reap greater return on investments compared to those accessing shared spaces). We have elaborated on this point in the manuscript, with relevant references **(Lines 194 – 199)**.

The work sows doubt about who will appropriate the value generated (platforms or consumers) through various references, this interesting issue remains unresolved throughout the work. This issue is very important in order to be able to establish international legislation for sharing platforms, which may be using international trade law as a reference and adapting it as much as possible.

Thanks for the comment. We do agree that the issue of value appropriation in sharing economy business models is a critical one. How value is appropriated, by which actors, and the implications of this for international legislation and trade law are interesting questions. However, we feel this is beyond the scope of our manuscript. However, we do indicate that the use of our framework and tool are of interest to legal experts regulating the sharing economy and related practices **(Lines 34, 70, 97)**. We hope you agree that this suffices.

The research of the literature must be more precise, critical and profound, that is, the concepts must appear at the beginning of the text, their definition must be precise and their analysis must be well structured.

Again, we have made strides to improve the structure of the document by introducing key concepts at the beginning of the text, including precise defintions (see previous point). We point the reviewer to the section *Background Literature*, which now includes elaboration on the sharing economy, social impacts of sharing platforms, and discussion on social sustainability and social impacts **(Lines 93 – 117, 147 – 157, 203 – 219)**. We have also restructured this section and added Table 1 **(Line 157)**. We suggest the additional text here seeks to clarify our approach, while engaging with the literature more critically and profoundly. Thanks for the suggestion!

**Introduction**
The introduction should be more general and introductory. Perhaps an overview of the “sharing economy” and what aspects have been previously addressed in the literature and what are the main contributions of the work (including here a summary of the methodology and its objectives).

In the *Introduction*, we have now provided a more general introduction to the sharing economy and the contestation surrounding the social impacts of sharing platforms **(Lines 37 – 60)**. In addition, we highlight the main objectives of our work and summarise the methodology **(Lines 78 – 87)**. We took effort to avoid duplication between the *Introduction*, *Background Literature*, and *Methods*.

The concepts “empower”, “trust”, “social cohesion”, “democratic cohesion”, ... should be discussed at the beginning but in detail, so from my point of view they should be located in a later section. They are concepts that arise a lot at work and are not grouped (the reader is lost when he wants to know that each concept has been previously said). On the other hand, I think that a good definition with a clear border for each concept, avoiding intersections, would add value to the work.

We agree with the reviewer and have made effort to improve upon their point. As already stated, we now introduce the four social aspects – trust, empowerment, inclusivity, and social justice – already in the *Introduction* **(Lines 78 – 83)**. In addition, we provide additional elaboration of these concepts in the *Background Literature*, including Table 1 with working definitions and relevant literature **(Lines 146 – 158)**. While we agree that there remains some contestation between concepts used in our manuscript, this is largely a result of existing literature. It was not our aim to resolve these conflicts in literature, but to propose a framework to support assessing the social impacts of sharing platforms. On this front, we feel we have improved our manuscript by introducing key concepts early on in the manuscript, as suggested, which are built upon successively throughout the text. We are grateful for this suggestion, as we think it ultimately enhances our manuscript!

The level of bibliographic review does not make it necessary to establish a different section of the introduction. The important thing in this part is to establish initial general references that raise the subsequent argumentation. If the contribution is the clarification of concepts and the discussion of the workshop, in order to establish indicators, it should be aroused here that these concepts are used fuzzily in literature but are important for management.

Thank you very much for the interesting comment. Respectfully, we would like to clarify that the contribution of our paper is not as much in clarifying the concepts, but rather in proposing a framework for assessing social impacts of sharing platforms. However, to address their point, we now offer definitions supported by extant litearture in order to bring clarity to the framework (see above points). Similarly, we discuss results and findings from the stakeholder workshop because they serve as input to the framework development, not to define concepts, per se.

However, to address your particular point, we have made additional effort to problematise the importance of defining these fuzzy concepts for managers **(Lines 93 – 101)**. We hope this suffices, in addition to our other efforts to enhance the structure and add relevant definitions early in the manuscript.

From my point of view lines, 101-106 can be the basis of the introduction.

We appreciate that this text resonated with the reviewer. We used this as inspiration to improve our *Introduction* as well as restructure the section containing *Background Literature*. Now, we feel the presentation of ideas and contribution are clearer in our manuscript, for example, considering **Lines 39 – 45, 78 – 83, 93 – 101**.

Line 109 I find hedonic motivations hard to justify.

Thanks for the comment. In our manuscript, the corresponding line references Belk (2010; 2014), who remains among the most cited researchers in the field. We stand by this reference and our point that the motivation among users affects the outcome (i.e. social impact) of sharing via a platform. However, to be more convincing and clear, we have elaborated further **(Lines 135 – 138)**.

**Social impacts of sharing platforms**
This section presents the justification of the concepts used in the search for the best collective management. This must be clear but it is difficult work. From my point of view the emergence of “sharing platforms” are a cause, not a consequence of social cohesion, increased confidence, ... although all this has to be justified well (line 126). Aspects such as inclusion or exclusion are in the air.

In general, the question of causality is a bit tricky. However, this is why we have taken the perspective of the sharing platform in developing our framework, to suggest that the social impacts are a consequence of sharing. We have formulated this now in **Lines 141 – 143, 148 – 152**. In addition, we add that we are focusing only on one direction, the social impact from sharing, not the impact of social behaviour on sharing. This is an interesting area for future research!

In addition, we have restructured the entire section relating to social impacts of sharing platforms. By introducing Table 1 **(Line 157)**, we hope to better justify our approach and provide more clear definitions to more concretely deal with the varying terms that were previously “in the air”.

The idea that “sharing platforms” link performance to skills and less to scale economies, and this is an aspect sociality integrative should be highlighted and developed. In this sense, education plays a key role in avoiding exclusion.

Thanks for the suggestion. We have further integrated this perspective into our manuscript, considering economies of scale **(Lines 108 – 111)**.

We agree with the reviewer that education plays a key role in avoiding social exclusion, for example. However, in this particular section, we seek to only provide an overview of the social impacts of sharing platforms discussed in literature. We present social inclusion/exclusion as part of our framework later in the manuscript, where we already mention the need for anti-discrimination training to avoid exclusion **(Line 832)**. We point the reviewer to this section of our manuscript, where we suggest it is more appropriate.

“Assessing social sustainability” must be included in the previous point.

We totally agree that the actual assessment of social sustainability should be included in our research. We have elaborated on this in a newly names section in the *Background Literature*, called *Social Sustainability and Social Impact Assessment* **(Lines 203 – 219)**. In addition, we raise this point for future research, as the focus of this paper is to develop an assessment tool that could be used in assessing social sustainability of sharing platforms **(Lines 973 – 987)**.

**Methodology**
This section should serve to clarify and specify the concepts already presented in the previous section, justifying the choice made according to the methodology. Some technique, a little more sophisticated, is missed to make ranking (for example AHP).

Thanks for the feedback. We will consider the analytical hierarchy process (AHP) for future stakeholder workshops. Unfortunately, we are unable to revisit our informants at this time and update the ranking presented in our manuscript. However, we feel this does not detract from our overal contribution, as the ranking did not inform or influence the final framework. It is simply an interesting data point, among many. We can also remove it, at the request of our reviewers.

New concepts should not appear here. Once the concepts have been chosen, the indicators for each concept must be justified. The number of concepts and indicators should be reduced and concreted.

Thank you very much for the suggestion. We have followed your advice and introduced the four aspects of the framework early on in the paper, ensuring no new concepts appear in the *Methodology*. Furthermore, our enhancements better justify the aspects selected in this study. The selection of our indicators is a result of our data analysis. This was a very thorough process, based on literature and our stakeholder workshop. We are comfortable with introducing the indicators (as new concepts) when we present the overall framework, as they represent the results of our data analysis. We are open to any specific suggestions by the reviewer to reduce or formulate the indicators differently. However, at this point, we are comfortable with the number and depth of indicators as a result of our data analysis.

Participation in governance must be clarified, which means and how it is done. Getting feedback from users has always been done, but that doesn’t mean they’re involved in governance.

Thanks for the suggestion. Quite right! Governance is not simply requesting feedback, but involving individuals or groups in the decision-making process. We have now elaborated on our understanding of governance at the first instance we mention the concept in the presentation of our framework **(Lines 595 – 598)**. In previous research (Curtis & Mont, 2020), we have elaborated on this extensively, inspired by Muñoz and Cohen (2018). Beyond this, we do not think it is vital to go into greater depth, as this is not the primary contribution of our manuscript.

It tries to cover a lot with little justification, such as talking about personal growth.
Problems arise from the limits of concepts, is the same “empowers” as “Stakeholder collaboration” or “inclusivity”? What are the differences?

Thanks for your comment. Indeed, the specific development and justification of every aspect or indicator is not described at length. However, we elaborate and justify our approach in the *Methodology* section, including the aspects **(Lines 290 – 298)** and indicators **(Lines 362 – 379)**. We reiterate this justification when presenting the results from the stakeholder workshop and introduce the social sustainability framework. In order to avoid significant overlaps, we judge the sum of provided justifications throughout the manuscript to be sufficient.

Various aspects and indicators are chosen based on literature and the workshop, as elaborated in the manuscript, including the indicator of personal growth. Please see the following reference for the importance of personal growth as a social indicator: Zhang, Tingting, Diego Bufquin, and Can Lu. “A Qualitative Investigation of Microentrepreneurship in the Sharing Economy.” International Journal of Hospitality Management 79 (2019): 148–57. 2)

Finally, the drafting should be reviewed, as an example, line 96 "We use the term user to include the actors involved...". Line 428 “ICT” has not been previously defined.

We have reviewed the document once more for minor grammatical errors and typos. In addition, we have had the document reviewed by a copyeditor prior to submission.

We thank the reviewer for catching the acronym, which was not previously defined. We have now defined this at the first appearance in the text.

Once again, we thank you for your feedback. We hope to have addressed all of your points in our revised manuscript!

**Reviewer #2:**

Thank you for the opportunity to read and comment on this submission. This paper first analyzes and describes the social impacts indicators of sharing platforms according to the existing literature, and then the key part of the paper is the opinions and analysis of the stakeholder seminar. Then, the results of the stakeholder seminar and the content of the literature are integrated, and 18 indicators included in four social aspects are determined to form the evaluation framework. All of them are narrated and have a literature basis. However, the following issues may be solved further.

The focus of this paper is to evaluate the social impacts of sharing platforms. The paper summarizes the indicators describing the social impacts of sharing platforms in terms of social sustainability. Why to study the indicators of social sustainability, the paper needs to explain the relationship between social impacts of sharing platforms and social sustainability, or point out the definition of social impacts of sharing platforms in this paper.

This is a great suggestion! Thank you! We have restructured the section *Background Literature* to support this elaboration **(Lines 35 – 138, 147 – 158)**. Furthermore, we have elaborated on the defintions of social sustainability, social impact, and their relationships **(Lines 203 – 219)**.

This paper classifies the stakeholders, but I still can't clearly see the impacts of sharing platforms on them or the relationship between them, whether positive or negative. I hope that this description may be added.

Thanks for the comment. Does the reviewer mean stakeholders (those that participated in our stakeholder workshop) or actors in the sharing economy (e.g. platform, users, and society)? Without understanding the full context of the suggestion, we are only able to partially address this comment. Firstly, considering the stakeholder categories – they expressed interest in assessing social impact of sharing platforms, having self-selected to attend our workshop. As we also asked them to consider their role as a citizen and/or user of sharing platforms, they may also be able to speak to the social impacts they have experienced or observed.

Secondly, considering the actors in the sharing economy, we have added a brief description of the impacts of sharing platforms on them in the section *Background Literature* **(Lines 148 – 156)**.

Beyond this, our analysis presented in the section *Social Sustainability Framework* and the tool presented in the section *Operationalising the Tool* clearly indicate the impacts on the users and society, when relevant, including indicators to assess these impacts for each actor. We hope this addresses the reviewer comment and welcome any further feedback to improve this area of our manuscript.

Resource users and resource owners are important participants in sharing economy. Citizens are the important group in the evaluation of social impact. Why these three groups are not included in workshop?

Thanks for your question. Indeed, we were aware of this potential limitation of our approach and therefore we already included this in the manuscript when discussing limitations **(Lines 964 – 966)**. As we stated, in order to reduce this limitation, we asked workshop participants to consider social impacts and social indicators from the perspective of their stakeholder category as well as users and citizens. To increase clarity, we also have added this to the relevant section of the *Methodology* **(Lines 318 – 319)**.

As the input from the stakeholder workshop is complemented with literature, which often included user and citizen perspectives, we hope this was an acceptable way to address this limitation.

According to the stakeholder's ranking of the four social aspects, the importance, grade or weight of the indicators has obtained certain results. Then in the fourth step of the paper structure, it is proposed to carry out data analysis, because the paper aims to provide measurable indicators to evaluate the social impacts of sharing platforms, the paper does not give the specific data analysis process, also do not enumerate the measurement results of the relevant data analysis, adding data analysis can show the rationality and scientificity of the system framework.

Thank you very much for the valuable comment. There are a few points to address here.

Firstly, the ranking of the social aspects by the stakeholder categories is an interesting data point, but does not inform the significance or weight of any aspects or indicators in our framework. In fact, we mention more than once that any specific weight should be based on the priorities of the user of the tool, as many of the indicators are context specific and their importance is subjective **(e.g. Lines 852 – 854, 922 – 923)**.

Secondly, we elaborate our own data analysis process in *Step Four* of the *Methodology* **(Line 362+)**. This process included the coding of the content from the stakeholder workshop as well as the literature from our narrative literature review, as already articulated in this section. Beyond this, the reviewer is correct in their observation, we did not collect primary data relating to measuring each aspect or indicator. As we state in the *Introduction*, obtaining access to this data from the platform and their users is challenging **(Lines 73 – 77)**. Therefore, the aim of our research was to develop a framework for assessing social impacts of sharing platforms, not to assess the impacts as such.

In future research, we intend to collaborate with one or more sharing platforms to collect the data and carry out an assessment of their social impact. We already indicate this in the *Discussion and* C*onclusion* section, when discussing future research **(Lines 973 –987)**.

This study proposes a framework to assess social impact of sharing platforms. Why did it assess from four dimensions (platform, resource owner, resource user, and the society). Can it represent the boundary of social impact?

Thanks for the question. In enhancing the section *Background Literature*, we have sought to make clearer the justification of these four actor groups. For example, we have included a description of these actors **(Lines 120 – 126)** as well as better justify why user motivation is important when assessing impact **(Lines 135 – 138)**. By examining the experiences and practices of these four actor groups, we comprehensively seek to assess the social impacts of sharing platforms. As such, the reviewer may see this as representing a sort of boundary.

The last point is the relationship between the cultural background and the indicators proposed in this paper. Whether the indicators proposed in the paper are applicable to the social impacts of sharing platforms in all cultural environments remains to be discussed. If the premise description of social and cultural background can be given, this paper will make greater contributions.

Thanks for the suggestion. We have more clearly stated in several places that the participants in the stakeholder workshop were primarily Swedish **(e.g. Lines 88, 300 – 301, 960 – 962)**. However, the literature review was all-encompassing, considering cultural contexts including the United States, China, Ghana, among others. Furthermore, we already discuss the need to consider social and cultural context in the section *Discussion and Conclusion* **(Lines 965 – 972)**. While the indicators will differ based on context and the priorities of those using the tool, we have developed and tested the tool with actors in Sweden and the Netherlands **(e.g. Lines 385 – 392)**, suggesting some compatibility among Western capitalistic cultures.

**Reviewer #3:**

In my opinion the manuscript is technically correct and comply with journal guidelines. The problem of sharing platform is new one and may be the object of multidisciplinary research. I would like to note a view the practice of writing about non-heterosexual human behaviours as a condition of alleged discrimination is not appropriate for the prestige of science, furthermore for the moral values and common good.

Thanks for reading our manuscript and providing positive feedback.
